# Effect of Molecular Weight and Nanoarchitecture of Chitosan and Polycaprolactone Electrospun Membranes on Physicochemical and Hemocompatible Properties for Possible Wound Dressing

**DOI:** 10.3390/polym13244320

**Published:** 2021-12-10

**Authors:** Maria Oviedo, Yuliet Montoya, Wilson Agudelo, Alejandra García-García, John Bustamante

**Affiliations:** 1 Grupo de Dinámica Cardiovascular, Centro de Bioingeniería, Universidad Pontificia Bolivariana, Medellín 050031, Colombia; mariac.oviedo@upb.edu.co (M.O.); wilson.agudelo@upb.edu.co (W.A.); john.bustamante@upb.edu.co (J.B.); 2 Laboratorio de Síntesis and Modificación de Nanoestructuras and Materiales Bidimensionales, Centro de Investigación en Materiales Avanzados, Chihuahua 31136, Mexico; alejandra.garcia@cimav.edu.mx

**Keywords:** nanoarchitecture, bilayer membrane, electrospinning, chitosan, hemocompatibility

## Abstract

Tissue engineering has focused on the development of biomaterials that emulate the native extracellular matrix. Therefore, the purpose of this research was oriented to the development of nanofibrillar bilayer membranes composed of polycaprolactone with low and medium molecular weight chitosan, evaluating their physicochemical and biological properties. Two-bilayer membranes were developed by an electrospinning technique considering the effect of chitosan molecular weight and parameter changes in the technique. Subsequently, the membranes were evaluated by scanning electron microscopy, Fourier transform spectroscopy, stress tests, permeability, contact angle, hemolysis evaluation, and an MTT test. From the results, it was found that changes in the electrospinning parameters and the molecular weight of chitosan influence the formation, fiber orientation, and nanoarchitecture of the membranes. Likewise, it was evidenced that a higher molecular weight of chitosan in the bilayer membranes increases the stiffness and favors polar anchor points. This increased Young’s modulus, wettability, and permeability, which, in turn, influenced the reduction in the percentage of cell viability and hemolysis. It is concluded that the development of biomimetic bilayer nanofibrillar membranes modulate the physicochemical properties and improve the hemolytic behavior so they can be used as a hemocompatible biomaterial.

## 1. Introduction

Tissue engineering (TI) has emerged as an alternative to restore and improve the function of an injured organ or tissue. TI is based on the union of three fundamental principles: (i) the development of three-dimensional membranes, (ii) the interrelation with bioactive molecules, and (iii) the functionalization with embryonic or adult cells [1]. Therefore, TI focuses on the development of biomaterials with architectures that emulate the extracellular matrix of the native tissue. It favors an active cellular interaction in the tissue regeneration process through the release of differentiation and growth factors and cell proliferation and migration. Finally, these factors allow the bio-integration of the biomaterial in the native tissue [2].

Within the biological processes, there is a biochemical, biophysical, and morphological essential interrelationship for the regeneration process. Therefore, three-dimensional membranes must exhibit characteristics such as biocompatibility, porosity, chemical anchorage sites, mechanical properties, and biodegradability, which allow integration and interconnection with the host tissue [3].

In this context, the electrospinning technique has been used for the development of fibrillar structures at micro- and nanometric scales. This has allowed the emulation of a morphological and functional behavior similar to the native extracellular matrix. At the morphological level, the fibers form an interconnected structure with an average diameter of 50 nm and an apparent pore size between 15–20 nm. With these characteristics are favored mechanical properties such as tensile and elastic resistance and hydrophilic and permeable properties that allow the structure to act as a biophysical ion-exchange filter. Therefore, the main advantage of this technique is the ability to generate multiscale fibrillar morphologies through the variation of parameters such as needle–collector distance, voltage, flow, or relative humidity. The above has generated interest in the development of microarchitectures for the biomedical field that favors biological, biocompatible, biodegradable, and hemocompatible characteristics [4,5,6].

Likewise, electrospinning allows the use of a wide variety of polymers of both natural and synthetic origin and polymeric mixtures, which improve the physicochemical properties of the fibrillar matrix. In this sense, chitosan as a natural polymer has exhibited adequate biological, chemical, and physical properties, which has made it a potential biomaterial in the health sector. Some research has used chitosan as a biomaterial. Chitosan favors hemocompatibility, thanks to the electrostatic interaction mediated by the positive charge density in the polysaccharide structure and the negative charge present in the cellular structures of erythrocytes. These properties favors the hemoconcentration of the coagulation factors that are activated to form a fibrin clot [7,8,9,10,11].

Similarly, the use of synthetic polymers such as polycaprolactone (PCL) has allowed obtaining scaffolds with mechanical and biodegradable characteristics. Despite lacking native biocompatibility, the mixtures with natural polymers decrease the solutions viscosity. This allows the fibers’ formation at the nano- and micrometer scale, providing a greater interaction between the fibrillar structure and the biological model [12,13,14].

On the other hand, the molecular weight of the polymers of natural origin plays an important role in the final biomedical application. The chain length of a polymer when interacting in a polymeric mixture can present changes in its chemical composition. This increases the active sites for the formation of biochemical interactions that improve the hydrophilic, mechanical, and morphological properties [15,16,17]. Likewise, the molecular weight affects the rheological properties of the polymeric mixtures such as viscosity, surface tension, and conductivity, which in synergy with the electrospinning parameters favor the stability of the Taylor cone and thus the formation of fibers without the presence of defects. This influence allows the fabrication of a scaffold with a defined microarchitecture that exhibits characteristics such as degradation rate, migration, adhesion, and cell proliferation. This is related to pore size, porosity, surface area, and surface chemical arrangement due to the higher molecular weight polymer chains, all in the function of favoring the regeneration of the native tissue [18,19].

It is important to mention that hemocompatibility has been studied from a chemical perspective, but it is necessary to know its effect as biomaterial with multiscale fibrillar morphology. This morphology could intervene in the deposition, fiber density, and patterns similar to tissue interweaving, and, in turn, in the biological response and coagulation cascade since a high fiber density should restrict the passage of erythrocyte and platelet-like blood cells to promote rapid fibrin formation and slow nutrient absorption [20].

Based on the above, the present work was oriented to the development of electrospun bilayer membranes composed of low and medium molecular weight chitosan and PCL, evaluating their physicochemical and hemocompatible properties.

## 2. Materials and Methods

### 2.1. Materials

Polycaprolactone (PCL) with a molecular weight of 80 kDa, low (ChLMW) and medium (ChMMW) molecular weight chitosan with a degree of deacetylation between 75–85% with a molecular weight between 50,000–190,000 and 190,000–310,000 Da, respectively, were acquired from Sigma Aldrich. Glacial acetic acid, 99.5% purity, and sodium chloride, pharmaceutical grade, were purchased from Panreac AppliChem (Barcelona, Spain). Formic acid with 99% purity and sodium hydroxide in lentils was purchased from Merck Millipore (Burlington, VT, USA).

### 2.2. Preparation of the Material

#### 2.2.1. Preparation of Working Solutions

Solutions of 12% *w/v* PCL, and 2% *w/v* ChLMW, ChMMW were prepared using a mixture of formic and acetic acid as a solvent in a volumetric ratio of 3:2. The resulting solutions were stirred previously for 24 h; then, the polymer mixture was made and stirred again for the same period.

#### 2.2.2. Electrospinning of the PCL/ChMMW and PCL/ChLMW Membranes

The PCL/ChLMW and PCL/ChMMW mixtures were deposited in a 5 mL glass syringe, and the electrospinning conditions varied according to the modifications in the parameters of needle–collector distance, flow rate, voltage, and volumetric concentration of the polymers (see Table 1). The final membranes consisted of electrospinning biocomposite from the parameters listed in Table 2, where they were electrospinning for a final time of 10 h.

### 2.3. Physicochemical Characterization of Biocomposite Membranes

#### 2.3.1. Scanning Electron Microscopy (SEM)

The morphological characteristics of the electrospinning materials were analyzed in a Jeol NeoScope JCM-600 plus and a Nova NanoSEM 200 scanning electron microscope, at an accelerating voltage of 10 and 15 kV, respectively. The micrographs were analyzed at magnifications of 10,000× and 100,000× using ImageJ^®^ software [21] to determine the average fiber diameter and apparent porosity. For this analysis, two micrographs were used for each type of sample, and 50 fiber diameters in each of them were measured.

Likewise, the polydispersity index (*PdI*) of the biocomposite membrane fibers was determined from the results obtained from the processing of the micrographs, using Equation (1).

(1) $$PdI={\left(\frac{\sigma }{\alpha }\right)}^{2}\;\;\;$$

where *σ* is the standard deviation of the fiber diameter distribution and *α* to the average filament diameter.

Therefore, *PdI* values close to 1 correspond to a larger diameter distribution and heterogeneous samples. For *PdI* values less than 0.1, the distribution is considered monodisperse [22].

#### 2.3.2. Fourier Transform Infrared Spectroscopy (FTIR)

The chemical analysis of the functional groups presents in the biocomposite electrospinning membranes was performed by Fourier transform infrared spectrophotometry with an attenuated total reflection module (FTIR-ATR) using a Nicolet iS50-FTIR spectrophotometer. The spectra were obtained at a resolution of 4 cm^−1^ by the accumulation of 32 scans in a wavelength between 4000–400 cm^−1^.

#### 2.3.3. Dynamic Mechanical Analysis (DMA)

Uniaxial static mechanical stress tests of biocomposite membranes with dimensions of 5.3 mm width × 9 mm length were performed through a dynamic mechanical analyzer (DMA Q800, TA Instruments, New Castle, DE, USA). The tests were carried out in force-controlled mode with a preload of 0.05 N with load increments of 0.2 N/min for the PCL control membranes and a preload of 0.001 N with load increments of 0.1 N/min for the PCL/ChLMW and PCL/ChMMW samples. Then, the tensile strength and Young’s modulus were calculated from the slope of the stress–strain curve.

#### 2.3.4. Contact Angle Wettability

To determine the hydrophilicity of the PCL, PCL/ChLMW, and PCL/ChMMW membranes, the contact angle was recorded using a goniometer model Oca 15 plus with a 12 V line voltage adjusted with an optical system. The test consisted of depositing a drop of water and blood plasma individually on the surface of the biocomposite membranes. For this analysis, three replicates were used for each type of sample, and frames were recorded for 2 min.

#### 2.3.5. Degradation

The rate of degradation of the membranes was evaluated to determine the weight loss of the biocomposites in interaction with fluids. PCL, PCL/ChLMW, and PCL/ChMMW samples with dimensions of 1.5 × 1.5 cm were used for the tests; then, their thickness was measured, and their weight is taken. Then, the membranes were immersed in 1 mL of saline solution with pH 5 (acid) and 10 (basic) and deionized water at pH 7.4 (neutral) during incubation periods of 4, 6, and 24 h; then, the weight was obtained in wet and dry. Finally, the percentage of weight loss was determined from Equation (2).

(2) $$\%Weight\;loss=\frac{Weight_{Dry\;1}-Weight_{Dry\;2}}{Weight_{Dry\;1}}\times 100\;\%$$

where $Weight_{\mathrm{Dry}\;1}\;$ corresponds to the initial dry weight of the PCL, PCL/ChLMW, and PCL/ChMMW samples, respectively. $Weight_{Dry\;2}\;$ is the final weight after interaction with the fluid.

#### 2.3.6. Static Permeability Test

To determine the resistance to flow through the microarchitecture of biocomposite membranes, the coefficients of hydraulic conductivity were obtained experimentally, through the adaptation of the ISO-7198 standard for biomedical devices [23]. For the test, a hydrostatic pressure column was used with a fluid height equivalent to the pressures supported by blood vessels in the outer layers of the skin (3, 5, and 10 mmHg). Then, the samples were placed in a sample holder and connected to one end of the pressure column, determining the mass of the working fluid (distilled water) that flows through the membrane for 1 min. With the results obtained, the hydraulic conductivity of the membrane was reported using Equation (3).

(3) $$K_{\mathrm{ISO}7198}=\frac{m_{f}}{\rho_{\mathrm{fluid}}\times \;A\;\times t}$$

where K_ISO7198_ is the hydraulic conductivity (mL/min∙cm^2^), $m_{f}$ is the mass of the working fluid (g), $\rho_{\mathrm{fluid}}$ is the density of the working fluid (g/mL), A is the area of the sample holder (cm^2^), and t is the collection time [min].

### 2.4. Biological Evaluation

#### 2.4.1. Hemolytic Test

**Figure 1:**
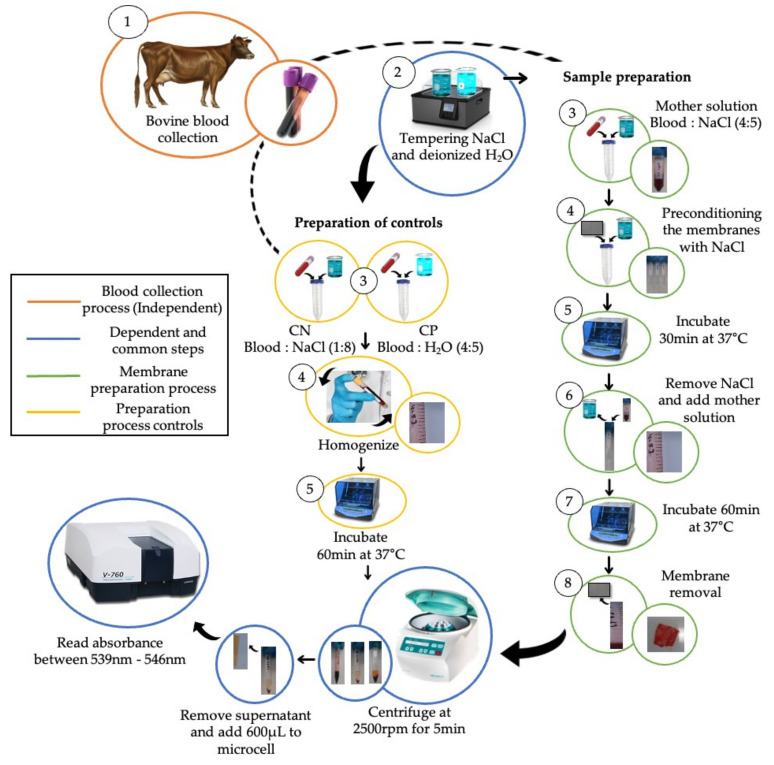
Methodological scheme to determine the hemolysis percentage of the PCL/ChLMW and PCL/ChMMW electrospun biocomposite membranes.

To perform the hemolysis assay (see Figure 1), the PCL/ChLMW and PCL/ChMMW samples were sterilized and then preconditioned when placed in tubes with saline solution for 30 min at 37 °C. A volumetric ratio, of 4:5 blood–saline stock solution, a 4:5 blood and deionized water positive control (CP), and a 1:8 blood and saline negative control (CN) were prepared as working solutions. After the preconditioning period, the biocomposite membranes were incubated for 60 min at 37 °C with a stock solution. Later, the samples were removed and centrifuged at 2500 rpm for 5 min. Finally, the absorbance of the membranes and controls were determined in a Bio Lambda 10 UV-VIS spectrophotometer at a wavelength of 545 nm. The percentage of hemolysis was determined using Equation (4).

(4) $$\%Hemolytic=\frac{A_{s}-A_{N}}{A_{p-A_{N}}}\times 100\%$$

where *A_s_* is the membrane absorbance, *A_N_* is the negative control absorbance, and *A_p_* is the positive control absorbance.

#### 2.4.2. In Vitro Model

The Swiss mouse embryonic NIH 3T3 fibroblast cell line was used to perform the assay. These were cultured in DMEM culture medium supplemented with 10% fetal bovine serum (FBS) and penicillin (100 U/mL)/streptomycin (100 μg/mL), and maintained in conditions of 5% CO_2_ and 95% O_2_ at 37 °C and 95% relative humidity. Once the 3T3 fibroblasts reached a cell confluence between 80–90%, they were subcultured, and the culture medium was changed every two days.

#### 2.4.3. Cell Viability

Cell viability was determined by the (3-(4,5-Dimethylthiazol-2-yl)-2,5-Diphenyltetrazolium Bromide) metabolic reduction (MTT) assay, which allowed determining the mitochondrial functionality of the treated cells and the possible cytotoxic effect of the electrospun membranes [24].

To determine the cell viability of 3T3 fibroblasts, indirect MTT of PCL, PCL/ChMMW, and PCL/ChLMW membrane leachates was performed, which were obtained from the interaction of each type of membrane previously washed with 70% *v/v* ethanol and sterilized with UV radiation by 30 min in DMEM medium without supplementation in incubation periods of 24, 48, and 72 h. Then, from the resulting solutions, 100% and 50% *v/v* solutions of the leachates of each type of membrane were prepared.

For this assay, 3.5 × 10^3^ cells/well of 3T3 fibroblasts were seeded in 96-well plates, treated with the leachates after 48 h, and left in interaction for 24 h. The cells were used as a positive control (C+) with 15% *v/v* hydrogen peroxide (H_2_O_2_) and as a negative control (CC) with no treatment. Each treatment was performed in triplicate. After 24 h, MTT was added; 4 h later, cold acidic isopropanol was added; and finally, the reading was performed in a multiplate reader at a wavelength of 570 nm. Cell viability was determined from the following equation:

(5) $$Cell\;viability=\frac{Sample\;optical\;density}{Control\;optical\;density}\times 100$$

### 2.5. Statistical Analysis

The results were statistically analyzed through a multifactorial ANOVA study, where the statistical influence was determined through the *p*-value < α premise, which was corroborated by the Shapiro–Wilk normality test and Bartlett homoscedasticity test, under the *p*-value > α hypothesis. In addition, the values obtained in the characterization techniques are expressed as the mean ± the standard deviation. Likewise, an *n* = 3 and a level of statistical significance with *p* < 0.05 were considered for each technique.

## 3. Results and Discussion

### 3.1. Scanning Electron Microscopy

**Figure 2:**
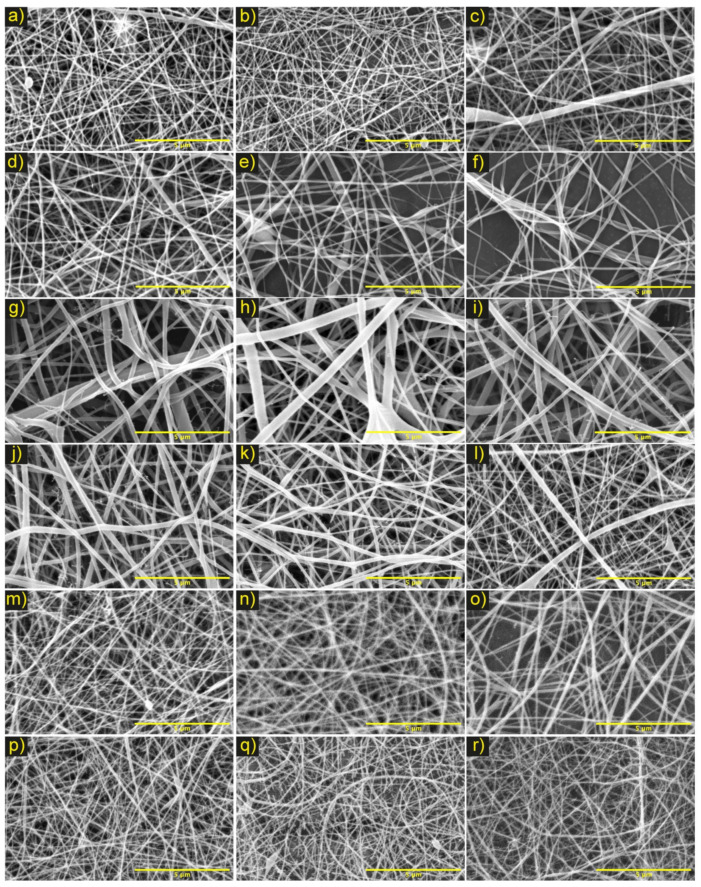
Micrographs of electrospinning PCL/ChLMW membranes under the parameters of the experimental design. Where combination No. 1 (**a**) 10 cm and 10 kV, (**b**) 15 cm and 10 kV, (**c**) 10 cm and 15 kV, (**d**) 15 cm and 15 kV, (**e**) 20 cm and 15 kV; combination No. 2 (**f**) 10 kV and 15 cm, (**g**) 11 kV and 15 cm; combination No. 3 (**h**) 11 kV and 10 cm, (**i**) 11 kV and 20 cm; combination No. 4 (**j**) (6:4) (PCL: CLMW), (**k**) (5:5) (PCL: CLMW), (**l**) (4:6) (PCL: CLMW), (**m**) (3:7) (PCL: CLMW) and combination No. 5 (**n**) 0.30 mL/h and 18 cm, (**o**) 0.30 mL/h and 25 cm, (**p**) 0.50 mL/h and 18 cm, (**q**) 0.50 mL/h and 20 cm, and (**r**) 0.50 mL/h and 25 cm. Scale bar, 5 μm.

**Figure 3:**
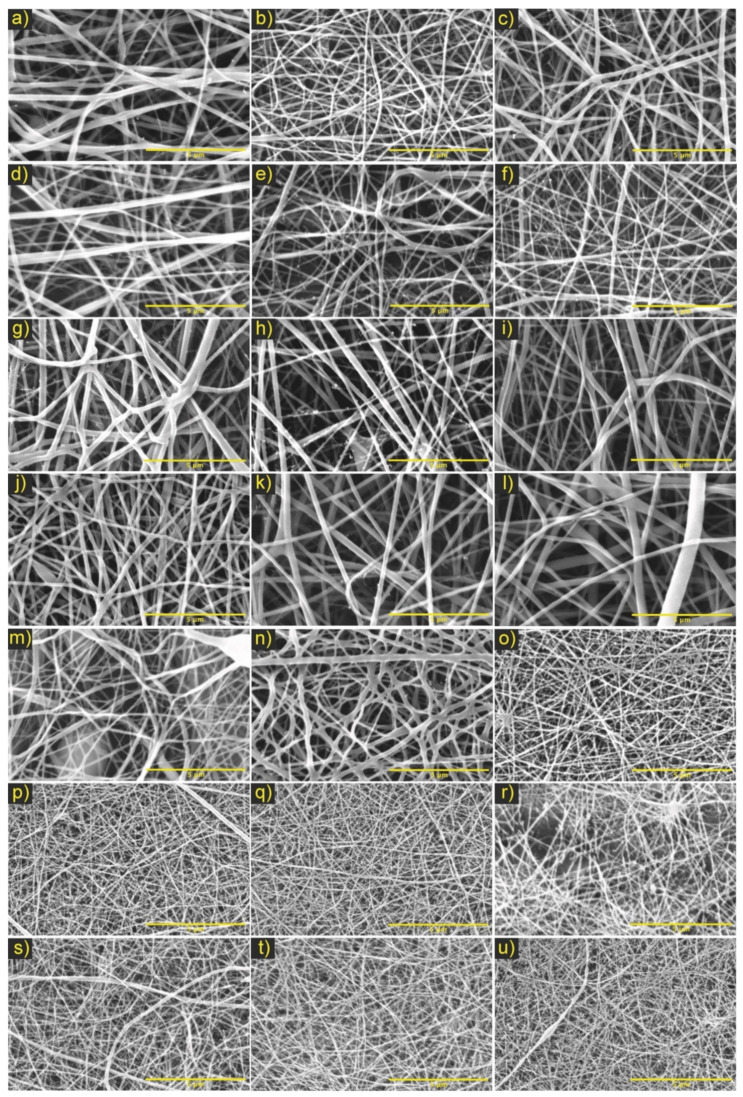
Micrographs of electrospinning PCL/ChMMW membranes under the parameters of the experimental design. Where combination No. 1 (**a**) 10 cm and y10 kV, (**b**) 15 cm and 10 kV, (**c**) 20 cm and 10 kV, (**d**) 10 cm and 15 kV, (**e**) 15 cm y15 kV, (**f**) 20 cm and 15 kV; combination No. 2 (**g**) 20 kV and 15 cm, (**h**) 20 kV and 18 cm, (**i**) 20 kV and 20 cm, (**j**) 22 kV and 15 cm, (**k**) 22 kV and 18 cm, (**l**) 22 kV and 20 cm; combination No. 3 (**m**) (6:4) (PCL: CMMW), (**n**) (5:5) (PCL: CMMW), (**o**) (4:6) (PCL: CMMW), combination No. 4 (**p**) 0.30 mL/h and 18 cm, (**q**) 0.30 mL/h and 20 cm, (**r**) 0.30 mL/h and 25 cm, (**s**) 0.50 mL/h and 18 cm and combination No. 5 (**t**) 0.50 mL/h and 20 cm, and (**u**) 0.50 mL/h and 25 cm. Scale bar, 5 μm.

Based on the results of the experimental design for the electrospinning process, the conditions were found that allowed obtaining PCL/ChLMW and PCL/ChMMW membranes (See Figure 2 and Figure 3) with continuous, homogeneous, defect-free fibers and fiber diameters close to the nanometer scale.

According to obtained results, it was evident that, in PCL/ChLMW membranes, the fibers were randomly oriented due to the type of fixed plate collector. The final fiber diameter values were observed between 60 and 150 nm (see Table 3), where the pearlized defects were attributed to the partial evaporation of the solvent, causing low collection of material and accumulation, as well as widening of fibers (see Figure 2a,c,e–h). On the other hand, it was evidenced that the formation of fibers with fewer defects presented at voltages between 10 and 11 kV, at flows between 0.3–0.5 mL/h, and distances between 18–25 cm (see Figure 2f–r). This behavior is because the voltage in combination with flow parameters and polymer concentration confer stability in the Taylor cone, causing the fiber diameter to decrease as distance increased [25]. Similarly, in combinations No. 1, 2, and 3, a behavior directly proportional to the fiber diameter and voltage was observed, together with a low viscosity of the solution, which caused the polymer mixture to be expelled in greater quantities and to accumulate, favoring an increase in the fiber diameter [26].

Additionally, in combination No. 2 for PCL/ChLMW membranes, increases in voltage were found to lead to material collection and increases in fiber diameters (see Figure 2f,g), behavior that could be attributed to an increase in the potential differential and lower viscosity of the solution that generates a greater amount of polymer solution expelled and deposited in the collector [27]. On the other hand, a decrease in the fiber diameter was evidenced (see Figure 2j–m) as the chitosan concentration increased. This is due to the polycationic nature of the chitosan, which influences the increase in conductivity and to a greater charge density in the solution generating a lengthening of the fiber and a decrease in its diameter [28]. Finally, in combination No. 5 (PCL/ChLMW), a decrease of the fiber diameters was observed as the flow increased, which could be related to a greater stretching of the fibers generated by the amount of polymer mixture expelled and the voltage applied to the system [29].

For the experimental designs of the PCL/ChMMW membranes, fibers with random orientation, that are continuous and uniform, with the presence of fine fibers that interconnect in larger fibers and average diameters of the final combinations between 33–90 nm were found (see Table 4). This phenomenon is attributed to the presence of chitosan in the sample, which, due to its molecular weight and concentration, increased the charge density due to its polycationic nature, generating a secondary division of the solution that influenced the formation of superimposed nanofibers on the fibers of greater diameter (see Figure 3a–l) [30].

Additionally, from the results of combinations No. 1 and 2, it was determined that the increase in fiber diameter concerning distance could be attributed to apparent solution viscosity, voltage variation, and changes in needle–collector distance, which generated instability in the formation of the Taylor cone [14]. Likewise, in combination No. 2, it was determined that, by increasing the concentration of the polymeric mixture, more homogeneously fused fibers were obtained, while increasing the needle–collector distance, efficient evaporation of the solvent, and absence of polymeric accumulation in the final sample were favored.

On the other hand, in the combination No. 3 of PCL/ChMMW samples (see Figure 3m–o), changes in the morphology of the fibers were observed as the chitosan concentration increased, which would be related to the change in the apparent viscosity and conductivity of the solutions to be electrospun [31,32]. Likewise, the instability of the Taylor cone influenced the formation of fibers with border irregularities and fiber thickening (see Figure 3m,n). Likewise, there was a decrease in the size of the fibers as the flow and concentration of the chitosan increased (see Figure 3p–s). This variation is due to the molecular composition of the chitosan and the differential of applied potential, which, by presenting positive charges around its chemical structure, favored the increase in conductivity and the stretching of the fibers [33].

From the morphological analysis of the PCL/chitosan samples, it was determined that the membranes with an ideal fibrillar microarchitecture for the development of bilayer membranes able of emulating a selective filter were Figure 2o,q for PCL/ChLMW and the membranes in Figure 3q,r for PCL/ChMMW.

**Figure 4:**
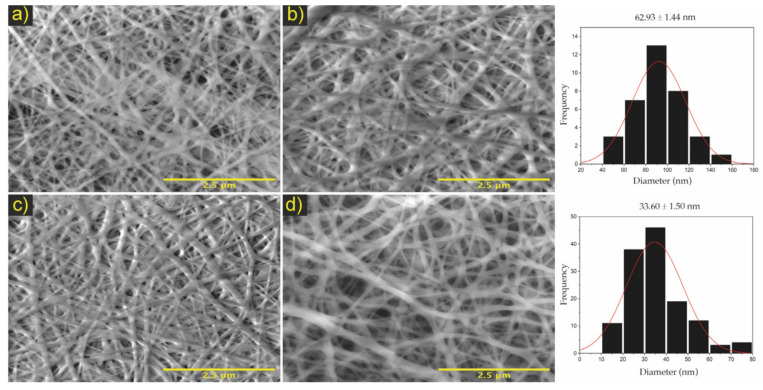
Micrographs the electrospinning bilayer membranes. Where (**a**) PA/PM/PB of PCL/ChLMW, (**b**) PB/PM/PA of PCL/ChLMW, (**c**) PA/PM/PB of PCL/ChMMW, (**d**) PB/PM/PA of PCL/ChMMW. On the right side, the histograms of the fiber distribution of the membranes are shown. Scale bar, 5 μm.

The membranes in Figure 4a,b presented a pore size of 39.2 and 21.9 nm, while Figure 4c,d had a pore area of 22.5 and 19.1 nm (see Table 5). Based on the results obtained, it was found that the apparent pore size is influenced by the synergy between the different electrospinning parameters, especially between the flow rate and the needle–collector distance.

Finally, it was determined that the PCL/ChMMW and PCL/ChLMW membranes presented polydispersity indexes lower than 0.1, which indicates that the fibers exhibit a monodisperse and homogeneous behavior, which was compared with the fiber diameter and standard deviation, where accuracy was obtained in the experimental data and delimitation in the fiber diameter ranges.

### 3.2. Fourier Transform Infrared Spectroscopy

**Figure 5:**
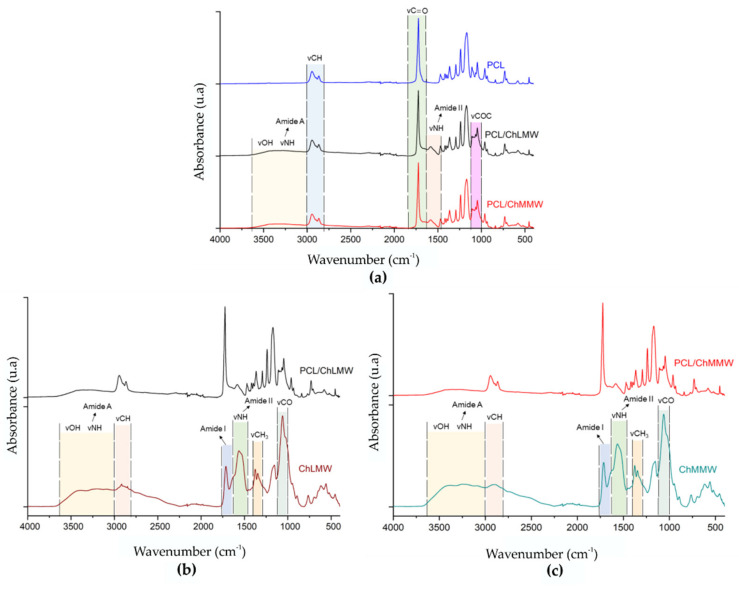
FTIR spectra of electrospinning scaffolds, where (**a**) control of PCL and PCL/ChLMW and PCL/ChMMW membranes, (**b**) control of ChLMW and PCL/ChMMW membranes, and (**c**) control of ChMMW and PCL/ChMMW membranes.

The infrared spectrum in Figure 5 shows the characteristic peaks of the functional groups presented in the structure of the PCL, ChLMW, and ChMMW. Figure 5a shows the presence of two spectral bands located at wavelengths of 2940 and 1723 cm^−1^, corresponding to the vibration of the groups -CH and -C=O of the PCL, which are present in less intensity in the membranes composed of PCL/ChLMW and PCL/ChMMW, which indicates the presence of the polycaprolactone in the final bilayer membranes [34,35].

The spectra of the biocomposite membranes were then compared with their respective chitosan controls to determine the chemical interaction between the two polymers (see Figure 5b,c). In the chitosan spectra, the absorption bands for amide I (1660–1630 cm^−1^) and amide II (1564 cm^−1^) were observed in the spectral region between 1700 and 1000 cm^−1^. Additionally, a vibration of the groups -CH_3_ and -CO was found at wavelengths of 1347 and 1062 cm^−1^, respectively [36]. Furthermore, the spectra showed bands between 3600–3000 cm^−1^ associated with the vibration of the -OH groups and the primary amines, and bands at a wavelength of 2870 cm^−1^, which is related to the stretching of the -CH. Similarly, band displacement corresponding to amide II at wavelengths between 1583–1582 cm^−1^ was evident for PCL/ChLMW and PCL/ChMMW spectra compared to chitosan controls, which was between 1564–1563 cm^−1^. Such behavior could be attributed to an interaction mediated by weak bonds such as hydrogen bonds between the carbonyl group of the PCL and the hydroxyl or amine group of the chitosan.

Considering that, in the range of 3600–3000 cm^−1^, the amine (-NH2) and hydroxyl (- OH) groups are located, which allow the identification of the interaction between both polymers, the area percentage of these functional groups was determined by deconvolution of the spectra of the PCL/ChLMW, PCL/ChMMW biocomposite membranes, and their respective controls (see Table 6). It was found that the percentage of primary amine groups decreased and there was an increase in the percentage of -OH of the composite membranes, which could be associated with chemical interactions between the carboxyl group of PCL and the amino and hydroxyl groups of chitosan [34], which could lead to the formation of ester and amide bonds. It should be mentioned that the spectra of the PCL/chitosan membranes showed a change in the frequency of the amide II group compared to the chitosan membrane, indicating that interaction at the molecular level between the polymeric components took place [37].

### 3.3. Dynamic Mechanical Analysis

**Figure 6:**
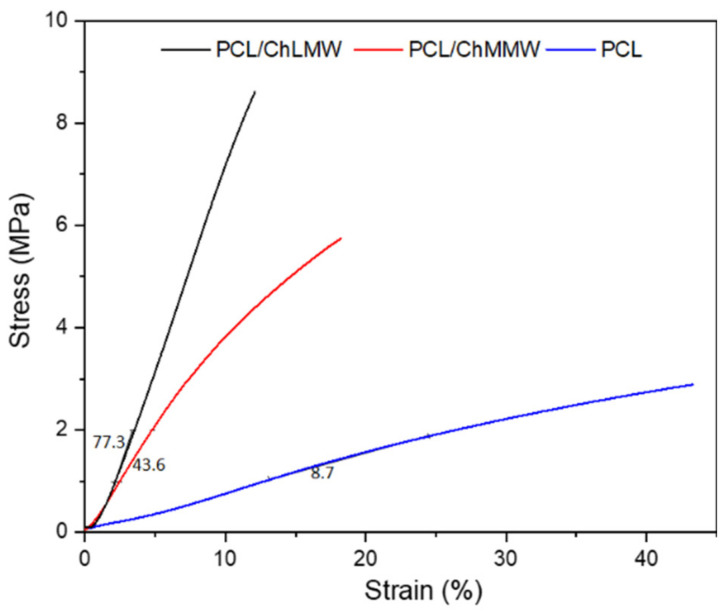
Stress–strain curve of PCL, PCL/ChLMW, and PCL/ChMMW control membranes.

Figure 6 shows how the incorporation of chitosan into the electrospinning membranes generated rigidity in the biocomposite material, with stress 13% and 18% for the PCL/ChLMW and PCL/ChMMW samples, respectively, compared to 47% ultimate tensile strength for the PCL control sample. This could be because the chitosan, due to its polycationic charge, increases the rigidity of the bilayer membrane from the interweaving of its chains with the synthetic polymer. Likewise, the difference in elongation between the chitosan samples is due to the increase in molecular weight and the presence of fiber diameters between 33 to 90 nm that make up the PCL/ChMMW membrane compared to the fiber diameter of the PCL/ChLMW membrane and PCL control, which have fiber sizes between 60 to 150 nm and 150 to 200 nm, respectively [38].

Likewise, it can be observed that Young’s modulus, as well as elongation percentages, was affected by the incorporation of chitosan in the microstructure of the membrane, increasing its value compared to that obtained for the PCL control. This behavior may be due to (i) the homogeneity and size of the fiber, where, for the PCL/ChLMW membranes, since they present larger fiber diameters in comparison to the PCL/ChMMW membrane, they can elongate more under the same type of applied force without losing their elastic property, which resulted in a Young’s modulus of 77.3 MPa compared to 43.6 MPa for the second type of membrane; (ii) the increase in pore size area and percentage of porosity, causing points of force concentration and therefore points of fracture [39]; (iii) the difference in Young’s modulus being possibly attributed not only to the presence and uniform distribution of chitosan in the PCL matrix but also to possible chemical or intermolecular interactions between the two polymers, as determined in the FTIR spectra, where a decrease in the percentage of primary amines in the biocomposite samples was evident [25].

### 3.4. Contact Angle Wettability

**Figure 7:**
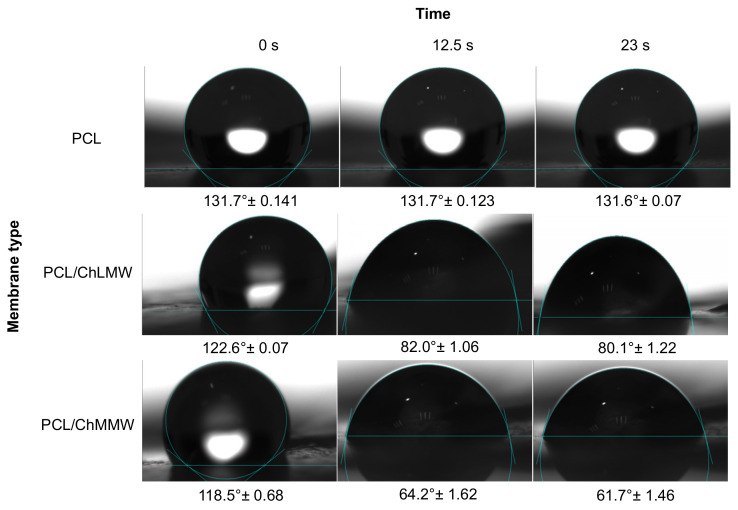
Contact angle for PCL, PCL/ChLMW, and PCL/ChMMW control membranes in interaction with deionized water.

**Figure 8:**
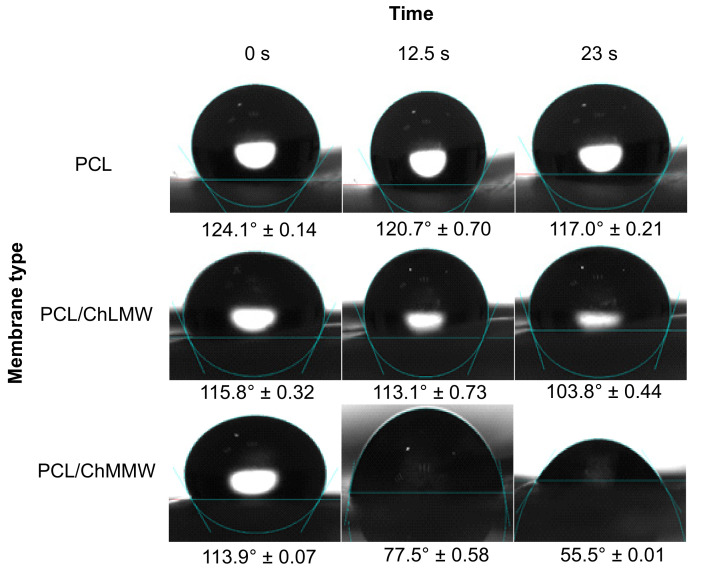
Contact angles for PCL, PCL/ChLMW, and PCL/ChMMW control membranes interacting with blood plasma.

Concerning the contact angle results obtained for the PCL, PCL/ChLMW, and PCL/ChMMW membranes (see Figure 7 and Figure 8), it was found that the PCL has a higher contact angle compared to the other types of membranes, due to the hydrophobic nature of this material, which does not allow the formation of bonding bridges with the molecules of the working solution. On the other hand, it was observed that the presence of chitosan in composite membranes decreases the contact angle, causing them to present a hydrophilic behavior, which is attributed to the presence of groups -OH in the chitosan side chains.

Additionally, it was found that PCL/ChMMW membranes had a lower contact angle in water and blood plasma compared to PCL/ChLMW samples. This may be because water absorption depends on both porosity and chitosan content. Although the porosity is similar between both membranes, the PCL/ChMMW sample has a larger pore size area, which allows the drop to permeate the scaffold structure easily. On the other hand, the possible structural accommodation of the chitosan chains on the hydrophobic structure of PCL results in obtaining a greater hydrophilic surface area that interacts with the working fluids, as a result of obtaining structures with greater porosity [40].

On the other hand, the presence of smaller diameter fibers in the PCL/ChMMW membranes gave rise to a greater area of contact with the environment that surrounds the material, while the liquid–solid interaction is greater being influenced by the size of the pore, which caused that when interacting with blood plasma a similar behavior will be presented.

When evaluating the behavior of both types of biocomposite membranes with blood plasma, a similar behavior to that found for water was observed, although there was a decrease in the contact angle values as a function of time. This behavior could be related to the presence of -OH groups in the side chains of the composite material, which interact with the protein structures present in the plasma.

### 3.5. Degradation

The presence of chitosan in the biocomposite membranes resulted in a greater weight loss when interacting with the three types of media, acid, neutral, and basic, compared to the PCL control membranes. These results are related to the hydrophilicity conferred by the chitosan in the scaffold structure, which provides anchorage points due to the -OH groups. On the other hand, PCL samples show a lower weight loss, which would be related to their hydrophobic compositional structure and the absence of points of interaction with fluids.

**Figure 9:**
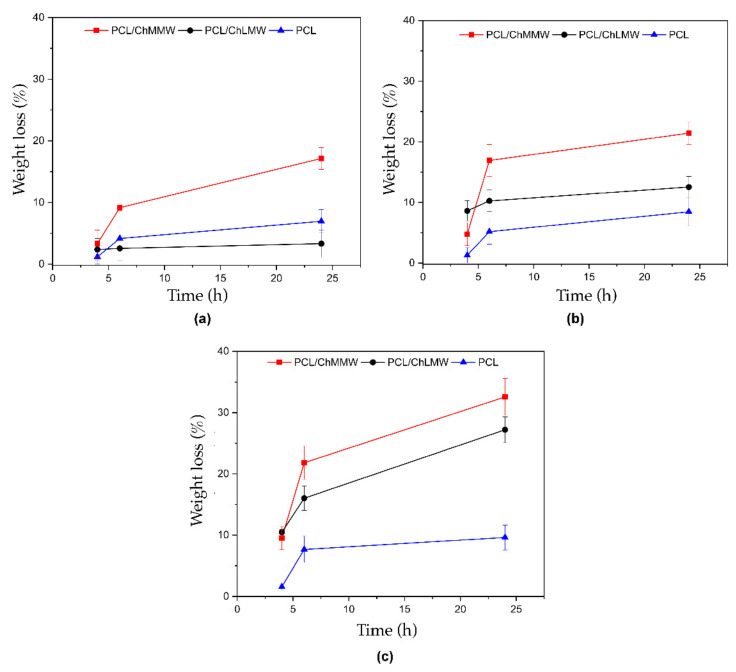
Weight loss versus time for electrospinning membranes of PCL, PCL/ChMMW, and PCL/ChLMW in interaction with (**a**) basic, (**b**) neutral, and (**c**) acids solutions.

The foregoing is evidenced in Figure 9a, where the PCL/ChMMW membrane in interaction with basic media exhibits a weight loss of 17.1%; such behavior could be due to the presence of hydrophilic groups such as amines in the chitosan chains. This characteristic reveals the level of interaction of the medium with the membrane since such biodegradability depends largely on the amount and nature of the intermolecular interactions of the chitosan chain. It should be noted that the weight loss in the PCL/ChLMW membranes may be due not only to the interaction that arises through hydrogen bonds between hydroxyl groups and amino groups but also to the diameter of the nanometer fiber, which favors a greater interaction area by increasing the surface contact area [41].

Previous research has shown that chitosan with deacetylation levels of 60% is soluble under neutral pH conditions. It has even been shown that the solubility of this polysaccharide under neutral pH conditions is achieved by controlling the degree of deacetylation and its molecular weight [40,41]. Such behavior was observed in Figure 9b, where the PCL/ChLMW and PCL/ChMMW membranes showed a higher mass loss compared to the PCL membranes, due to the hydrophobic character of the synthetic polymer.

On the other hand, the solubility of chitosan in acidic media (see Figure 9c) is due to the protonation of the amino groups present in the polysaccharide chain resulting in a high density of positive charges. Furthermore, the composite membranes presented a higher weight loss when interacting with acidic media, exhibiting values of 32.5% PCL/ChMMW, 27.2% PCL/ChLMW, and 9.6% PCL, which could be attributed to the presence of carbonyl groups in the PCL side chains, and the protonation of amino groups present in the ChMMW chains that generate an increase in polarity and electrostatic repulsions [42]. For this reason, the difference between the percentages of weight loss of the PCL/ChLMW and PCL/ChMMW membranes are due to the difference in the chitosan chain length that favors greater availability of amino groups.

### 3.6. Static Permeability Test

The permeability for electrospinning membranes depends on the capacity of the used technique to obtain porous structures, which, in this specific case, is around 30% for the three types of materials, which allows for a large surface area per unit of volume for each membrane. Likewise, it depends on the area of the pore sizes, homogeneity of the sample, and its hydrophilicity.

**Figure 10:**
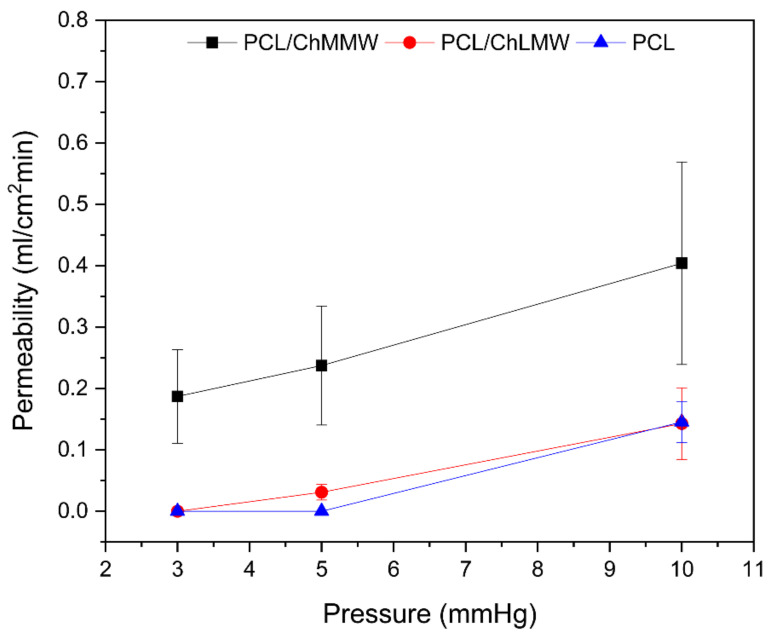
Permeability of electrospinning membranes of PCL, PCL/ChLMW, and PCL/ChMMW.

As show in Figure 10, the PCL membrane presented a lower permeability than the PCL/ChLMW and PCL/ChMMW samples, due to larger diameters and cross-linking of fibers, which makes it difficult for the solution to pass and allows its permeability only at one pressure of 10 mmHg. Likewise, it was evidenced that the PCL/ChMMW membranes presented higher permeability than the PCL/ChLMW membranes and with PCL control, because of a greater apparent pore area, fiber diameters, and the presence of —OH groups, attributed to the molecular weight of the chitosan present in the scaffold [35,43].

### 3.7. Hemolytic Test

**Figure 11:**
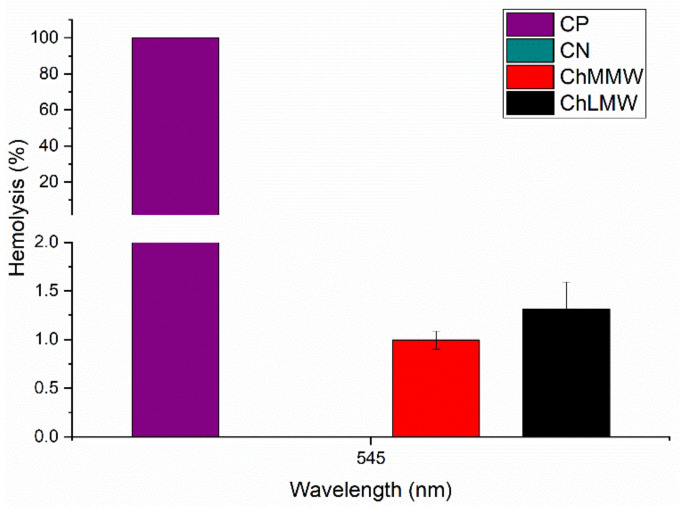
Hemolysis percentage for PCL/ChLMW and PCL/ChMMW biocomposite membranes, positive control and negative control.

The hemocompatibility assay of the PCL/ChMMW and PCL/ChLMW membranes revealed hemolysis percentages of 1.1% and 1.3%, respectively (see Figure 11). These results indicate that biocomposite scaffolds exhibit a hemocompatible nature in accordance with the ISO 10993−4 [44], where such a hemolysis percentage should be less than 5% for application in medical devices [45,46].

Similarly, the difference in the percentages of hemolysis between the PCL/ChLMW and PCL/ChMMW scaffolds can be attributed to smaller fiber sizes that affect the percentage decrease, due to the fact that a greater interaction area allows accommodation and interaction of cellular structures in the scaffold [43,47]. Likewise, a higher percentage of -OH groups in the PCL/ChMMW membranes resulted in greater anchorage points that favor hydrophilicity with the blood, avoiding the generation of lysis of the blood cells [48].

### 3.8. Cell Viability

**Figure 12:**
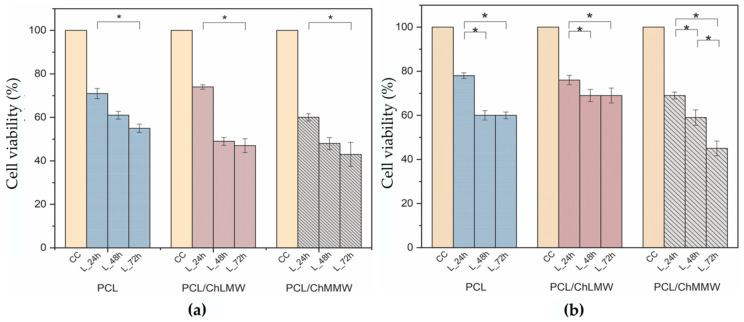
Cell viability of 3T3 fibroblasts upon interaction with PCL, PCL/ChMMW, and PCL/ChLMW membrane leachates, (**a**) 100% *v/v* treatment, (**b**) 50% *v/v* treatment. * Show the statistical significance of *p* < 0.05.

Figure 12 showed a decrease in the percentage of cell viability of 3T3 fibroblasts when interacting with leachates at 100% *v/v* and 50% *v/v* of electrospun membranes at three incubation periods of 24, 48, and 72 h. Likewise, it was observed that the 100% *v/v* leachates in comparison with the 50% *v/v* leachates generated a decrease in the cell viability percentage in all the evaluation periods, presenting a statistically significant effect on the viability of the fibroblasts, which could be attributed to the presence of free radicals resulting from the interaction of the polycaprolactone and chitosan with the biological model [49].

On the other hand, in Figure 12a, a decrease in cell viability of the fibroblasts was observed for each electrospun membrane in a time-proportional manner, presenting a statistically significant difference between the initial and final interaction period. This behavior may be due to the level of degradation exhibited by the membranes in neutral medium, which show a mass loss of 8% for PCL, 12% for PCL/ChLMW, and 21% for PCL/ChMMW.

Then again, Figure 12b shows a decrease in the cell viability of the PCL/ChMMW membranes in all the leachate evaluation periods, presenting a statistically significant difference between each time; however, the PCL and PCL/ChLMW membranes show a decrease in the percentage of viability in the 24 h period and a stabilization in the 48 h leachates. This behavior may be related to the dilution of the leachates and the length of the polymeric chain, which influences the number of active sites of the polymer to interact with the cell [50].

Importantly, the percentage of viability was lower in the PCL/ChMMW membrane for both treatments. In general, the decreases in cell viability of 3T3 fibroblasts could be attributed to the leachate exposure time, and the type of membrane due to the morphology of the material, since the PCL/ChMMW membrane presents smaller fiber sizes and an increase in the contact surface area, thus releasing a higher concentration of leachate. This behavior is related to the results obtained in the degradation test. In addition, by varying the length of the polymeric chain of the chitosan contained in the membranes, an increase in amino and hydroxyl groups occurs, which, when interacting with a neutral medium (pH ≈ 7.4), could decrease the viability of fibroblasts due to the protonation of functional groups and increase in free radicals that cause oxidative stress in the cellular model [36,51].

## 4. Conclusions

In this study, it was possible to electrospin solutions of PCL/ChLMW and PCL/ChMMW at volumetric ratios of 4:6 obtaining fiber diameters below 150 nm. It was found that the incorporation of chitosan in electrospun membranes increases the homogeneity of the solution and decreases the fiber diameter. The PCL membranes showed a diameter of 217 nm, while the PCL/ChMMW membrane showed decreases of 86 and 33 nm and the PCL/ChLMW membrane of 141 and 62 nm, for high and low porosity layers, respectively. On the other hand, a decrease in the pore area from 95 nm^2^ was obtained for PCL membranes until an average pore area of 39 and 21 nm^2^ for PCL/ChMMW and 22 and 19 nm^2^ for PCL/ChLMW membranes in the layers of high and low porosity, respectively.

It was evidenced that morphological characteristics such as fiber diameter and pore size influenced the increase in Young’s modulus from 8.7 MPa for the PCL membrane until 43.6 and 77.3 MPa for the PCL/ChMMW and PCL/ChLMW membranes. Furthermore, the incorporation of chitosan increased the polar anchoring points, increasing the hydrophilicity of the membranes. This characteristic affected the hemolytic behavior of the samples due to a greater contact angle and anchoring points that favored the interrelation of the material with blood cells. A decrease in the percentage of hemolysis of 100% for the positive control to percentages of 1.1% and 1.3% for PCL/ChMMW and PCL/ChLMW membranes was obtained.

Finally, we are concluded that the PCL/ChMMW membrane exhibited behavior similar to the intrinsic characteristics of the native extracellular matrix, due to a greater contact surface area and the ability to absorb fluids, which makes this type of three-dimensional biomaterials an alternative as a wound-dressing.

## Figures and Tables

**Table 1:** Design of experiments for the development of electrospinning PCL/ChLMW and PCL/ChMMW membranes.

	Combination	Volumetric Relation (*v*/*v*)	Parameters		
			Voltage (kV)	Flow Rate (mL/h)	Needle–Collector Distance (cm)
PCL/ChLMW	1	PCL/ChLMW (7:2)	10 15	0.05	10 15 20
	2	PCL/ChLMW (7:2)	10 11	0.30	15
	3	PCL/ChLMW (7:2)	11	0.30	10 20
	4	PCL/ChLMW (6:4); (5:5); (4:6); (3:7)	11	0.30	20
	5	PCL/ChLMW (4:6)	11	0.30 0.50	18 20 25
PCL/ChMMW	1	PCL/ChMMW (7:2)	10 15	0.05	10 15 20
	2	PCL/ChMMW (7:2)	20 22	0.50	15 18 20
	3	PCL/ChMMW (6:4); (5:5); (4:6); (3:7)	22	0.50	15
	4	PCL/ChMMW (4:6)	22	0.30 0.50	18 20 25

**Table 2:** Electrospinning parameters to obtain PCL/ChMMW and PCL/ChLMW biocomposite membranes with high (A) and low (B) porosity layer.

Membrane Type	Layer	Volumetric Relation (*v*/*v*)	Parameters		
			Voltage (kV)	Flow Rate (mL/h)	Needle–Collector Distance (cm)
PCL/ChMMW	A	(4:6)	22	0.30	25
	B	(4:6)	22	0.30	20
PCL/ChLMW	A	(4:6)	11	0.30	25
	B	(4:6)	11	0.50	20

**Table 3:** Summary design of experiments for the development of electrospinning PCL/ChLMW membranes.

Combination	Volumetric Relation (*v*/*v*)	Variable Parameters			Response Parameter
		Voltage (kV)	Flow Rate (mL/h)	Needle-Collector Distance (cm)	Fiber Diameter (nm)
1	PCL/ChLMW (7:2)	10 15	0.05	10 15 20 10 15 20	79.76 ± 1.30 76.09 ± 1.36 ---* 106.32 ± 1.34 109.57 ± 1.33 122.27 ± 1.41
2	PCL/ChLMW (7:2)	10 11	0.30	15	68.77 ± 1.44 185.51 ± 1.34
3	PCL/ChLMW (7:2)	11	0.30	10 20	131.88 ± 1.43 137.28 ± 1.38
4	PCL/ChLMW (6:4); (5:5); (4:6); (3:7)	11	0.30	20	121.43 ± 1.60 128.82 ± 1.40 77.24 ± 1.55 72.90 ± 1.39
5	PCL/ChLMW (4:6)	11	0.30 0.50	18 20 25 18 20 25	101.47 ± 1.32 77.24 ± 1.55 141.22 ± 1.53 82.17 ± 20.18 62.93 ± 1.44 89.75 ± 30.99

* Non-optimal parameters for fiber generation.

**Table 4:** Summary design of experiments for the development of electrospinning PCL/ChMMW membranes.

Combination	Volumetric Relation (*v*/*v*)	Variable Parameters			Response Parameter
		Voltage (kV)	Flow Rate (mL/h)	Needle-Collector Distance (cm)	Fiber Diameter (nm)
1	PCL/ChMMW (7:2)	10 15	0.05	10 15 20 10 15 20	217.33 ± 70.0 127.80 ± 1.40 177.14 ± 67.0 185.26 ± 1.53 110.62 ± 1.45 141.30 ± 1.43
2	PCL/ChMMW (7:2)	20 22	0.50	15 18 20 15 18 20	27.91 ± 29.2 203.05 ± 60.2 232.60 ± 90.6 171.64 ± 1.20 220.16 ± 1.22 277.68 ± 1.55
3	PCL/ChMMW (6:4); (5:5); (4:6); (3:7)	22	0.50	15	156.21 ± 66.2 139.46 ± 53.0 99.62 ± 1.39 ----*
4	PCL/ChMMW (4:6)	22	0.300.50	18 20 25 18 20 25	66.56 ± 1.35 33.60 ± 1.50 86.08 ± 1.27 56.93 ± 1.52 55.78 ± 1.40 83.21 ± 1.52

* Non-optimal parameters for fiber generation.

**Table 5:** Porosity and apparent pore size for PCL/ChMMW and PCL/ChLMW biocomposite membranes.

Material Type	Layer	Electrospinning Parameters	Fiber Diameter (nm)	Porosity (%)	Pore Size (nm^2^)	PdI
PCL/ ChMMW	A	22 kV; 0.30 mL/h; 25 cm	86.08 ± 1.27	31 ± 2.47	39.2 ± 1.76	0.008
	B	22 kV; 0.30 mL/h; 20 cm	33.60 ± 1.50	29 ± 0.70	21.9 ± 3.11	0.017
PCL/ ChLMW	A	11 kV; 0.30 mL/h; 25 cm	141.22 ± 1.53	31.6 ± 1.13	22.5 ± 0.77	0.010
	B	11 kV; 0.50 mL/h; 20 cm	62.93 ± 1.44	30 ± 3.53	19.1 ± 1.34	0.008

**Table 6:** Percentage area of ChLMW, PCL/ChLMW, ChMMW, and PCL/ChMMW membrane functional groups.

Functional Group	ChLMW	PCL/ChLMW	ChMMW	PCL/ChMMW
	Area (%)			
-NH_2_	82.04	52.23	50.17	38.13
-NH	---	---	---	---
-OH	17.95	47.77	49.82	61.87

## Data Availability

The data presented in this study are available on request from the from the corresponding author.

## References

[1] Estrada C., Paz A.C., López L.E. (2009). Ingeniería de tejido óseo: Consideraciones básicas. Rev. EIA Esc. Antioq..

[2] Zarei M., Samimi A., Khorram M., Abdi M.M., Golestaneh S.I. (2021). Fabrication and characterization of conductive polypyrrole/chitosan/collagen electrospun nanofiber scaffold for tissue engineering application. Int. J. Biol. Macromol..

[3] Poddar D., Jain P., Rawat S., Mohanty S. (2021). Influence of varying concentrations of chitosan coating on the pore wall of polycaprolactone based porous scaffolds for tissue engineering application. Carbohydr. Polym..

[4] Surucu S., Sasmazel H.T. (2016). Development of core-shell coaxially electrospun composite PCL/chitosan scaffolds. Int. J. Biol. Macromol..

[5] He X.X., Zheng J., Yu G.F., You M.H., Yu M., Ning X., Long Y.Z. (2017). Near-Field Electrospinning: Progress and Applications. J. Phys. Chem. C.

[6] Elahi M.F., Lu W. (2013). Core-shell Fibers for Biomedical Applications—A Review. J. Bioeng. Biomed. Sci..

[7] Li L., Du Y., Yin Z., Li L., Peng H., Zheng H., Yang A., Li H., Lv G. (2019). Preparation and the hemostatic property study of porous gelatin microspheres both in vitro and in vivo. Colloids Surf. B Biointerfaces.

[8] Rafique A., Zia K., Zuber M., Tabasum S. (2016). International Journal of Biological Macromolecules Chitosan functionalized poly (vinyl alcohol) for prospects biomedical and industrial applications: A review. Int. J. Biol. Macromol..

[9] Ahmed S., Sheikh J., Ali A. (2018). A review on chitosan centred scaffolds and their applications in tissue engineering. Int. J. Biol. Macromol..

[10] Usman A., Zia M., Zuber M., Tabasum S., Rehman S. (2016). Chitin and chitosan based polyurethanes: A review of recent advances and prospective biomedical applications. Int. J. Biol. Macromol..

[11] Muxika A., Etxabide A., Uranga J., Guerrero P., Caba K. (2017). Chitosan as a bioactive polymer: Processing, properties and applications. Int. J. Biol. Macromol..

[12] Ekram B., Abdel-Hady B.M., El-Kady A.M., Amr S.M., Waley A.I., Guirguis O.W. (2017). Optimum parameters for the production of nano-scale electrospun polycaprolactone to be used as a biomedical material. Adv. Nat. Sci. Nanosci. Nanotechnol..

[13] Zhang H., Zhao C., Zhao Y., Tang G., Yuan X. (2010). Electrospinning of ultrafine core/shell fibers for biomedical applications. Sci. China Chem..

[14] Kalwar K., Sun W., Li D., Zhang X., Shan D. (2016). Coaxial electrospinning of polycaprolactone chitosan: Characterization and silver nanoparticles incorporation for antibacterial activity. React. Funct. Polym..

[15] Karsli B., Caglak E., Prinyawiwatkul W. (2021). Effect of high molecular weight chitosan coating on quality and shelf life of refrigerated channel catfish fillets. Lwt.

[16] Susanto H., Robbani M.H., Istirokhatun T., Firmansyah A.A., Rhamadhan R.N. (2020). Preparation of low-fouling polyethersulfone ultrafiltration membranes by incorporating high-molecular-weight chitosan with the help of a surfactant. S. Afr. J. Chem. Eng..

[17] Babii O., Wang Z., Liu G., Martinez E.C., van Drunen Littel-van den Hurk S., Chen L. (2020). Low molecular weight chitosan nanoparticles for CpG oligodeoxynucleotides delivery: Impact of molecular weight, degree of deacetylation, and mannosylation on intracellular uptake and cytokine induction. Int. J. Biol. Macromol..

[18] Park B.K., Um I.C. (2018). Effect of molecular weight on electro-spinning performance of regenerated silk. Int. J. Biol. Macromol..

[19] Ko J., Jun S., Lee J.K., Lee P.C., Jun M.B.G. (2015). Effects of Molecular Weight and Temperature on Fiber Diameter of Poly(ε-caprolactone) Melt Electrospun Fiber. J. Korean Soc. Manuf. Technol. Eng..

[20] Gaston E., Fraser J.F., Xu Z.P., Ta H.T. (2018). Nano- and micro-materials in the treatment of internal bleeding and uncontrolled hemorrhage. Nanomed. Nanotechnol. Biol. Med..

[21] Ferreira T., Rasband W. (2012). ImageJ User Guide. ImageJ Fiji.

[22] Barham H.P., Harvey R.J. (2016). Hemostatic Materials and Devices Hemostatic Sinus Skull base Materials Vasoconstrictors Topical Agents. Otolaryngol. Clin..

[23] ISO-International Organization for Standardization (2016). ISO 7198: 2016 Cardiovascular Implants and Extracorporeal Systems—Vascular Prostheses—Tubular Vascular Grafts and Vascular Patches.

[24] Nga N.T.H., Ngoc T.T.B., Trinh N.T.M., Thuoc T.L., Thao D.T.P. (2020). Optimization and application of MTT assay in determining density of suspension cells. Anal. Biochem..

[25] Fadaie M., Mirzaei E., Geramizadeh B., Asvar Z. (2018). Incorporation of nanofibrillated chitosan into electrospun PCL nanofibers makes scaffolds with enhanced mechanical and biological properties. Carbohydr. Polym..

[26] Li Z., Wang C. (2013). Effects of Working Parameters on Electrospinning. One- Dimensional Nanostructures: Electrospinning Technique and Unique Nanofibers.

[27] Juncos Bombin A.D., Dunne N.J., McCarthy H.O. (2020). Electrospinning of natural polymers for the production of nanofibres for wound healing applications. Mater. Sci. Eng. C.

[28] Jia Y.T., Gong J., Gu X.H., Kim H.Y., Dong J., Shen X.Y. (2007). Fabrication and characterization of poly (vinyl alcohol)/chitosan blend nanofibers produced by electrospinning method. Carbohydr. Polym..

[29] Merchante R., Giménez E., Sahuquillo O. (2016). Análisis y Optimización de Parámetros de Proceso Para La Obtención de Fibras Poliméricas Tipo Core-Shell Mediante Electrospinning Coaxial.

[30] Nirmala R., Il B.W., Navamathavan R., El-Newehy M.H., Kim H.Y. (2011). Preparation and characterizations of anisotropic chitosan nanofibers via electrospinning. Macromol. Res..

[31] Karim M., Fathi M., Soleimanian-Zad S. (2020). Incorporation of zein nanofibers produced by needle-less electrospinning within the casted gelatin film for improvement of its physical properties. Food Bioprod. Process..

[32] Wang X.X., Yu G.F., Zhang J., Yu M., Ramakrishna S., Long Y.Z. (2021). Conductive polymer ultrafine fibers via electrospinning: Preparation, physical properties and applications. Prog. Mater. Sci..

[33] De Vrieze S., Westbroek P., Van Camp T., De Clerck K. (2010). Solvent system for steady state electrospinning of polyamide. J. Appl. Polym. Sci..

[34] Wan Y., Lu X., Dalai S., Zhang J. (2009). Thermophysical properties of polycaprolactone/chitosan blend membranes. Thermochim. Acta.

[35] Wang Y., Young T., Wang T. (2019). Investigating the effect of chitosan / polycaprolactone blends in differentiation of corneal endothelial cells and extracellular matrix compositions. Exp. Eye Res..

[36] Dimzon I.K.D., Knepper T.P. (2015). Degree of deacetylation of chitosan by infrared spectroscopy and partial least squares. Int. J. Biol. Macromol..

[37] Malheiro V.N., Caridade S.G., Alves N.M., Mano J.F. (2010). New poly(ε-caprolactone)/chitosan blend fibers for tissue engineering applications. Acta Biomater..

[38] Martínez G., Matos M., Sabino G.M., Urbina de Navarro C., Barrios C., Taddei A., Sajo C., Arnal M., Müller A. (2008). Estudio de una mezcla binaria biodegradable: Policaprolactona/quitina. Rev. Iberoam. Polímeros.

[39] Sánchez Cepeda Á.P. (2016). Preparación y caracterización de membranas poliméricas electrohiladas de policaprolactona y quitosano para la liberación controlada de clorhidrato de tiamina. Cienc. En. Desarro..

[40] Wu J., Liao C., Zhang J., Cheng W., Zhou N., Wang S., Wan Y. (2011). Incorporation of protein-loaded microspheres into chitosan-polycaprolactone scaffolds for controlled release. Carbohydr. Polym..

[41] Macea R.B., De Hoyos C.F., Montes Y.G. (2015). Síntesis y propiedades de filmes basados en quitosano/lactosuero Synthesis and film properties of chitosan and whey. Polímeros.

[42] Schmid B.C., Rezniczek G.A., Rolf N., Saade G., Gebauer G., Maul H. (2013). Uterine Packing with Chitosan-Covered Gauze for Control of Postpartum Hemorrhage. Am. J. Obstet. Gynecol..

[43] Miguel S.P., Moreira A.F., Correia I.J. (2019). Chitosan Based-Asymmetric Membranes for Wound Healing: A Review. Int. J. Biol. Macromol..

[44] ISO-International Organization for Standardization (2002). ISO 10993-4. Biological Evaluation of Medical Devices—Part 4: Selection of Tests for Interactions with Blood.

[45] Hemamalini T., Vikash N., Brindha P., Abinaya M., Dev V.R.G. (2020). Comparison of Acid and Water-Soluble Chitosan Doped Fibrous Cellulose Hemostat Wet Laid Nonwoven Web for Hemorrhage Application. Int. J. Biol. Macromol..

[46] Pardo-Castaño C., Bolaños G. (2019). Solubility of Chitosan in Aqueous Acetic Acid and Pressurized Carbon Dioxide-Water: Experimental Equilibrium and Solubilization Kinetics. J. Supercrit. Fluids..

[47] Sasmal P., Datta P. (2019). Tranexamic Acid-Loaded Chitosan Electrospun Nanofibers as Drug Delivery System for Hemorrhage Control Applications. J. Drug Deliv. Sci. Technol..

[48] Du X., Liu Y., Wang X., Yan H., Wang L., Qu L., Kong D., Qiao M., Wang L. (2019). Injectable hydrogel composed of hydrophobically modified chitosan/oxidized-dextran for wound healing. Mater. Sci. Eng. C.

[49] Yin J., Xu L. (2020). Batch preparation of electrospun polycaprolactone/chitosan/aloe vera blended nanofiber membranes for novel wound dressing. Int. J. Biol. Macromol..

[50] Zhang L., Dong Y., Zhang N., Shi J., Zhang X., Qi C., Midgley A.C., Wang S. (2020). Potentials of sandwich-like chitosan/polycaprolactone/gelatin scaffolds for guided tissue regeneration membrane. Mater. Sci. Eng. C.

[51] Saatcioglu E., Ulag S., Sahin A., Yilmaz B.K., Ekren N., Inan A.T., Palaci Y., Ustundag C.B., Gunduz O. (2021). Design and fabrication of electrospun polycaprolactone/chitosan scaffolds for ligament regeneration. Eur. Polym. J..

